# Robot‐assisted radical prostatectomy in patient with previous intersphincteric resection for rectal cancer

**DOI:** 10.1002/iju5.12793

**Published:** 2024-10-01

**Authors:** Naoki Imasato, Shugo Yajima, Ryo A Ogasawara, Minoru Inoue, Kohei Hirose, Ken Sekiya, Madoka Kataoka, Yasukazu Nakanishi, Hitoshi Masuda

**Affiliations:** ^1^ National Cancer Center Hospital East Chiba Japan

**Keywords:** colorectal cancer, colorectal surgery, prostate cancer, prostatectomy

## Abstract

**Introduction:**

There are often opportunities to consider treatment strategies for synchronous or metachronous prostate cancer with colorectal cancer. Performing robot‐assisted radical prostatectomy for prostate cancer following previous rectal cancer surgery in cases involving anal‐preserving surgeries such as low anterior resection or intersphincteric resection can be challenging because of the possibility of adhesions.

**Case presentation:**

A 74‐year‐old man who had undergone laparoscopic intersphincteric resection for rectal cancer was diagnosed with prostate cancer. The patient desired to undergo robot‐assisted radical prostatectomy. During surgery, we observed the absence of Denonvillier's fascia on the dorsal surface of the prostate, the intestinal anastomosis was distal to the vesicourethral anastomosis, and the rectum was replaced with a peristaltic sigmoid colon with minimal adhesions.

**Conclusion:**

Periprostatic conditions vary depending on previous rectal surgical approaches. It is crucial to confirm the previous surgical approach for rectal cancer when performing robot‐assisted radical prostatectomy following rectal cancer surgery.

**Abbreviations & Acronyms**
CRC: colorectal cancer
CT: computed tomography
DRE: digital rectal examination
DVF: denonvillier fascia
ISR: intersphincteric resection
LAR: low anterior resection
MRI: magnetic resonance imaging
PCa: prostate cancer
PI‐RADS: Prostate Imaging and Reporting Data System
PSA: prostate‐specific antigen levels
RARP: robot‐assisted radical prostatectomy

**Keynote message**

- We performed robot‐assisted radical prostatectomy for prostate cancer in a patient who had undergone intersphincteric resection for rectal cancer.
- Previous treatment for rectal cancer may have led to a different periprostatic situation.
- We performed robot‐assisted radical prostatectomy in a patient following intersphincteric resection, with minimal adhesions observed during the procedure.
- It is crucial to confirm the previous surgical approach to rectal cancer and prepare a meticulous preoperative plan when performing robot‐assisted radical prostatectomy following rectal cancer surgery.

## Introduction

The increasing incidence of PCa and CRC has led to an increase in oncological patients.^1^, ^2^, ^3^ PCa can be synchronous or metachronous in patients with CRC.^4^, ^5^ Simultaneous RARP and rectal cancer surgery have been performed.^5^, ^6^, ^7^, ^8^ Conversely, performing surgery for metachronous PCa in patients who have previously undergone rectal cancer surgery can be challenging due to adhesions.

Anal preservation surgeries such as LAR and ISR are increasingly common in patients with lower rectal cancer.^9^ Previous rectal cancer surgeries in which the anastomosis was situated dorsal to the prostate can complicate RARP.

We present the case of a patient who underwent RARP following ISR for rectal cancer, which has never been reported before. Surprisingly, despite the patient's history of ISR, intraoperative findings revealed minimal adhesions between the digestive tract and posterior aspect of the prostate.

## Case presentation

**Fig. 1:**
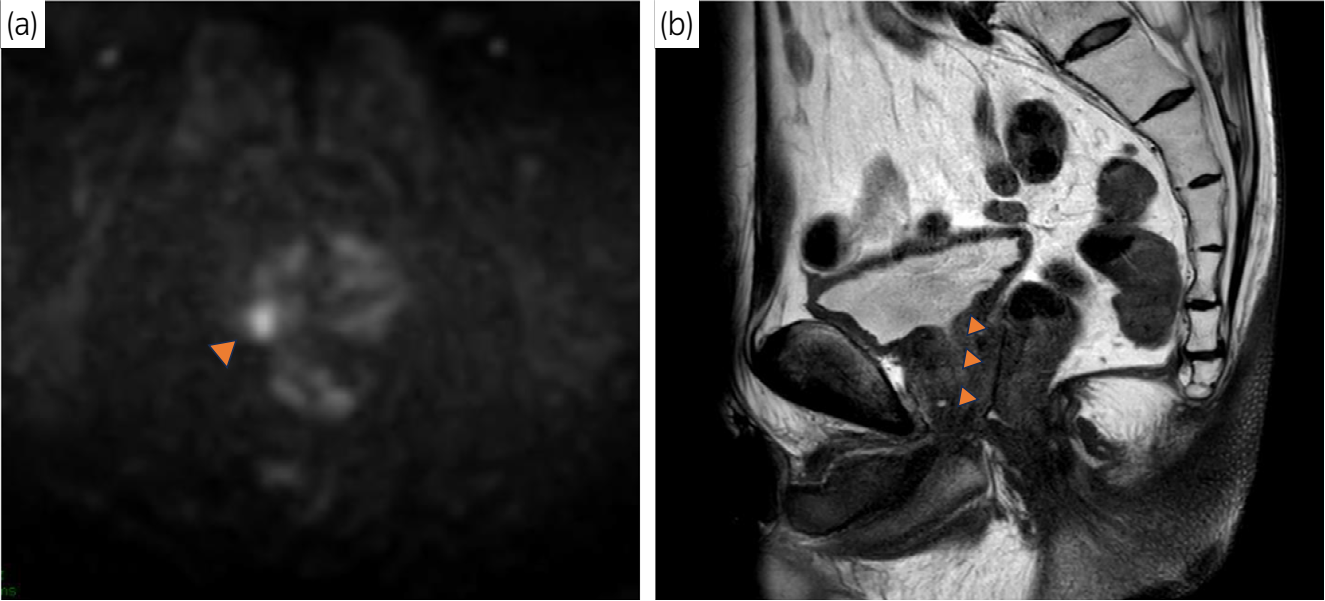
(a) Axial diffusion‐weighted MRI revealed prostate cancer lesion with a PI‐RADS category 4 in the right peripheral zone of the prostate. (b) Sagittal T2‐weighted MRI showed the prostate and its dorsal bowel were in contact, with no fat in between.

A 74‐year‐old Japanese man with a 20‐year history of laparoscopic ISR for rectal cancer was incidentally diagnosed with PCa during a follow‐up for rectal malignancy. The patient was referred to our department with elevated PSA of 9.06 ng/mL. Preoperative DRE revealed a firm nodule on the right lobe of the prostate. MRI revealed PI‐RADS 4 lesion in the right peripheral zone of the prostate (Fig. 1a). It also demonstrated an absence of fat between the prostate and dorsal bowel (Fig. 1b). Both CT and bone scans showed no metastatic lesions. Trans‐perineal prostate biopsy revealed prostatic adenocarcinoma with a Gleason score of 4 + 3. Of the 12 biopsy cores, prostate cancer was detected in 3 cores: 2 from the right lobe and 1 from the left lobe. We diagnosed localized PCa at clinical stage T2aN0M0. After the diagnosis of PCa, the patient requested RARP treatment.

**Fig. 2:**
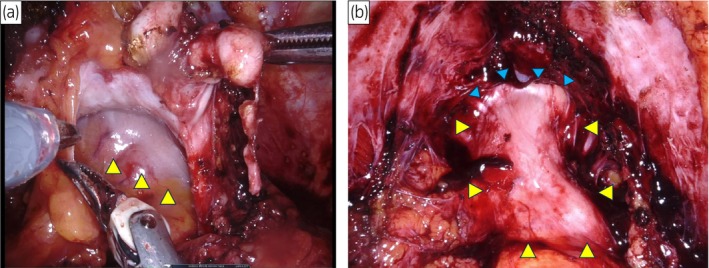
(a) There was little adhesion on the dorsal side of the prostate. DVF was not present, and the mobilized sigmoid colon (yellow arrowheads) was present. (b) Surface of sigmoid colon after prostatectomy. Peristaltic sigmoid colon (yellow arrowheads) and coloanal anastomosis line (blue arrowheads) were observed.

RARP was performed using the Da Vinci Xi surgical system (Intuitive Surgical Inc., Sunnyvale, CA, USA) with a 6‐port intraperitoneal approach. The patient was placed in the lithotomy position for the procedure. During surgery, we found an absence of DVF on the dorsal surface of the prostate, with minimal adhesions (Fig. 2a). The rectum was absent and replaced by a peristaltic sigmoid colon. Additionally, a coloanal anastomotic line was observed at the end of the sigmoid colon (Fig. 2b). The first layer of the posterior reconstruction was not performed because of the absence of DVF. No pelvic lymph node dissection was performed. The operative time was 175 min, console time was 95 min, and the estimated blood loss was 10 mL.

Urethrocystography was performed on postoperative day 6 confirmed the absence of leakage at the vesicourethral anastomosis site, and the patient was discharged on postoperative day 8. No severe postoperative complications were observed. At the 6‐month postoperative follow‐up, the patient achieved pad‐free urinary continence and maintained undetectable serum PSA levels. Pathological examination revealed a diagnosis of adenocarcinoma predominantly in the right lobe, classified as pT2a, with negative surgical margins.

## Discussion

There are various types of rectal cancer surgeries for rectal cancer, including anal preservation surgeries, such as LAR and ISR, and nonpreserving surgeries, such as abdominoperineal resection and Hartmann's procedure. ISR, an anal‐preserving surgery, is a surgical technique used to preserve sphincter function, mainly in cases of low rectal cancer located within 5 cm from the anal verge.^10^, ^11^, ^12^, ^13^ This surgery is performed by retaining part or all of the external anal sphincter. The usual procedure for ISR involves dissection of the rectum, including the DVF.^7^ In rectal cancer surgery, there are several optimal layers of dissection, some sparing the DVF.^14^, ^15^, ^16^

In this case, the DVF was absent on the dorsal surface of the prostate, and there was little adhesion. We consider two possible reasons for the little adhesion observed. First, the anastomotic line was caudal to the prostate. Adhesions can be expected with LAR, in which the anastomosis is closer to the prostate. Another reason may be the serosal surface of the sigmoid colon, which is presumed to prevent adhesion.

RARP after rectal cancer surgery is difficult because of the possibility of adhesions. One of the major challenges in prostate surgery after previous treatments for rectal cancer is the presence of periprostatic and intra‐abdominal adhesions. Intra‐abdominal adhesions have traditionally been considered a contraindication for minimally invasive radical prostatectomy.^17^ Periprostatic adhesions can complicate the identification of anatomical planes, especially during the dissection of the seminal vesicle and endopelvic fascia and may compromise the perioperative and functional outcomes of prostatectomy.

Recently, CRC survivors have increasingly required PCa treatment.^5^ There are reports that patients with CRC, especially those younger than 55 years of age, have an increased risk of developing secondary primary PCa.^3^, ^5^ With the expanding indications for minimally invasive surgery, it is relatively common for RARP to be performed in patients who have undergone previous pelvic surgery. However, RARP is usually excluded from PCa treatment after rectal cancer surgery due to the fear of intestinal injury. Alternative treatment options include radiotherapy and brachytherapy. Management of PCa after rectal cancer surgery is challenging for urologists. Nevertheless, we decided to perform RARP because of familiarity with several cases of salvage RARP for radiation‐recurrent PCa.^18^ For patients with rectal malignancies who have already received chemoradiation therapy, readministration may not be feasible if PCa develops later. Therefore, the techniques for performing RARP after rectal cancer surgery are necessary. Preoperatively, we must prepare for adhesions and develop strategies to manage them. In our clinical setting, patients with a history of rectal cancer surgery who underwent RARP were placed in the lithotomy position to undergo DRE. In addition, we used a transrectal ultrasound scan to confirm the position of the rectum when operating close to it. We also reviewed previous approaches to rectal cancer surgery.

Although several simultaneous RARP and rectal surgeries have been reported, reports on RARP in patients diagnosed with metachronous PCa after rectal cancer surgery are insufficient. It remains unclear whether the absence of adhesions in this case was coincidental or a phenomenon specific to RARP following ISR. There is a need for further data collection to better understand the impact of various surgical procedures, such as ISR, LAR, and Hartmann's procedure, on the outcomes of RARP. Given the complexities of performing prostatectomy after rectal cancer surgery, preoperative imaging to assess adhesions is essential. While we used conventional MRI, cine MRI could offer more detailed visualization, aiding in better surgical planning and outcomes. Moreover, this is the first report of RARP after ISR; therefore, minimal adhesion cannot be guaranteed in all cases. Further information regarding RARP after rectal surgery is expected to accumulate in the future.

## Conclusion

It is necessary to confirm the previous surgical approach for rectal cancer and prepare a meticulous preoperative plan for RARP after rectal cancer surgery. In addition, collecting and sharing more information on RARP following previous rectal cancer treatment is essential.

## Author contributions

Naoki Imasato: Conceptualization; data curation; formal analysis; methodology; project administration; resources; software; visualization; writing – original draft; writing – review and editing. Shugo Yajima: Conceptualization; data curation; project administration; supervision; validation; visualization; writing – original draft; writing – review and editing. Ryo A Ogasawara: Conceptualization; data curation; resources. Minoru Inoue: Data curation. Kohei Hirose: Formal analysis; software. Ken Sekiya: Data curation. Madoka Kataoka: Methodology; project administration; visualization. Yasukazu Nakanishi: Project administration; supervision; validation; writing – original draft; writing – review and editing. Hitoshi Masuda: Conceptualization; funding acquisition; project administration; supervision; validation; visualization; writing – original draft; writing – review and editing.

## Conflict of interest

The authors declare that they have no conflict of interest.

## Approval of the research protocol by an Institutional Reviewer Board

N/A.

## Informed consent

N/A.

## Registry and the Registration No. of the study/trial

N/A.
